# Depressive Symptomatology Is Associated With Self‐Reported Impaired Postural Balance in Older Adults: A Cross‐Sectional Study in Primary Care in Brazil

**DOI:** 10.1002/gps.70081

**Published:** 2025-04-28

**Authors:** Thiago Vinicius Nadaleto Didone, Catarina Costa Boffino, Nadine Seward, Carina Akemi Nakamura, Illora Aswinkumar Darbar Shimozato, Ricardo Araya, Tim J. Peters, Marcia Scazufca

**Affiliations:** ^1^ Departamento de Psiquiatria Faculdade de Medicina FMUSP Universidade de Sao Paulo Sao Paulo Brazil; ^2^ Departamento de Saúde Coletiva Centro de Ciências da Saúde Universidade Estadual de Londrina Londrina Brazil; ^3^ Instituto de Psiquiatria Hospital das Clinicas da Faculdade de Medicina HCFMUSP Universidade de Sao Paulo Sao Paulo Brazil; ^4^ Health Service and Population Research Institute of Psychiatry, Psychology and Neuroscience King's College London London UK; ^5^ Secretaria de Saúde da Prefeitura Municipal de Guarulhos Guarulhos Brasil; ^6^ Bristol Dental School University of Bristol Bristol UK

**Keywords:** balance complaint, Brazil, cross‐sectional study, depression, falls, impaired postural balance, older adult, primary health care

## Abstract

**Objectives:**

Age‐related balance deficits and depression are common among older people and challenging for public health. However, the association between postural imbalance and depression has scarcely been investigated in large samples, especially in low‐ and middle‐income countries (LMICs) whose populations are ageing rapidly. Here, we estimated the prevalence of postural imbalance and examined its association with depressive symptomatology among older adults living in a socioeconomically deprived area of Brazil.

**Methods:**

The analysis used screening data from the PROACTIVE cluster randomised controlled trial—specifically, socioeconomic, demographic and health information from individuals aged 60 years and older registered at one of 20 primary health clinics in Guarulhos and who provided complete data for our analyses. Self‐reported postural imbalance was the outcome and it was assessed with a single question. Participants who reported postural imbalance were asked about the number of falls they had experienced in the previous 6 months. The prevalence of postural imbalance and postural imbalance with or without falls was estimated. Depressive symptomatology was assessed using the Patient Health Questionnaire‐9 (PHQ‐9). The association between depressive symptomatology (PHQ‐9 score ≥ 10) and three ordered categories of the outcome (no postural imbalance, postural imbalance without falls and postural imbalance with falls) was investigated using adjusted mixed‐effects ordered logistic regression.

**Results:**

The study included 2999 individuals. Postural imbalance was reported by 1183 participants (39.4%; 95% confidence interval: 37.7%, 41.2%), comprising 792 non‐fallers and 391 fallers (26.4% and 13.0% of all participants, respectively). For participants with depressive symptomatology, the odds of having postural imbalance with or without falls versus not having postural imbalance is 2.88 (95% confidence interval: 2.44, 3.40) times that of participants without depressive symptomatology. Likewise, for participants with depressive symptomatology, the odds of having postural imbalance with falls versus having postural imbalance without falls combined with not having postural imbalance is 2.88 (95% confidence interval: 2.44, 3.40) that of participants without depressive symptomatology.

**Conclusions:**

Postural imbalance is a common occurrence in a vulnerable older population in Brazil. Importantly, we found that depressive symptomatology was associated with increased odds of having postural imbalance and postural imbalance with falls experienced in the previous 6 months. Notwithstanding our analyses' exploratory nature, these issues should receive greater attention in primary care practice and research.

**Summary**

- The relationship between depression and impaired postural balance remains unclear. Few studies were carried out with low‐income populations.
- We evaluated self‐reported impaired postural balance in a large sample of older adults registered in primary care clinics living in socioeconomically deprived areas of Brazil.
- Almost 40% of our sample had postural imbalance, a third of whom had experienced at least one fall in the previous 6 months.
- Older adults with depressive symptomatology were more likely to report postural imbalance and postural imbalance with falls than those without depressive symptomatology.

## 1 Introduction

Postural imbalance affects approximately 20% of the older population in the United States, half of whom seek medical care and a quarter of whom are unable to perform basic activities of daily living [1]. In Brazil, a study conducted in the municipality of São Paulo found a 16.3% prevalence of older individuals with balance disorders [2]. The most critical consequence of impaired postural balance is falls [3], which represent a major public health problem with high healthcare costs for the family, community and society [4]. Falls occur in 26.5% of older adults worldwide [5] and have harmful consequences such as injuries, bruises, fractures, reduced independence, loss of autonomy and even death [6]. In 2019, 1.6% of deaths in people aged 70 years and older were due to falls, and 69% occurred in low‐ and middle‐income countries (LMICs) [7]. From 2000 to 2020, public hospitals in Brazil recorded more than 1.7 million fall‐related admissions of people aged 60 years or over, representing an expenditure of 2.3 billion in Brazilian Real (BRL) for the Brazilian Unified Health System (SUS) [8].

Adequate functioning of the balance system allows the body to make corrections and adjustments to posture when necessary [9]. As people age, this system is often impaired, affecting standing, leaning, stepping, walking, mobility and transfer, which increases the occurrence of postural imbalance and falls [10, 11]. Besides age, depression also negatively influences balance. As a result, older people with depression have greater postural sway [12], slower gait speed [13], and longer stepping reaction time [14] than those without depression because this disorder may impair processes that contribute to maintaining posture, such as executive function [15, 16], attention [12] and neuromuscular function [17].

Depression has been consistently found to be a risk factor for falls [18, 19]. In contrast, there is a paucity of epidemiological information on the association between depression and impaired postural balance. The studies that have examined this association in large samples of community‐dwelling older people have shown mixed results. Some found an association between depression and postural imbalance [20, 21, 22], while others did not [2, 23, 24]. In addition, data from the English Longitudinal Study of Aging (ELSA) showed this to be a bidirectional association [22]. To the best of our knowledge, only two studies have investigated this association in Brazil, and neither found evidence for it [2, 24].

Investigations of postural balance problems in older people with depression, and in particular the identification of which individuals are most affected by these conditions, are urgently needed, especially in LMICs, where the number of older people is growing rapidly, and there are limited initiatives to provide mental health care and fall prevention services [25, 26]. Therefore, using a large sample of older adults living in socioeconomically deprived areas of Brazil, this study aimed to: (a) estimate the prevalence of individuals with postural imbalance; (b) calculate the prevalence of individuals with postural imbalance *with* and *without* falls experienced in the previous 6 months; and (c) investigate the association between depressive symptomatology and postural imbalance. We hypothesised that postural imbalance is more frequent in people with depressive symptomatology than those without it and, among those with postural imbalance, those with depressive symptomatology present more falls than those without depressive symptomatology.

## 2 Materials and Methods

### 2.1 Study Design and Sample

This cross‐sectional study used data collected during the screening assessment for the PROACTIVE cluster randomised controlled trial (*N* = 3356). PROACTIVE was a task‐shared, collaborative care psychosocial intervention to improve recovery in older people with depressive symptomatology registered with 80 Family Health Teams (FHTs) in the 20 participating *Unidades Básicas de Saúde (UBSs)* in socioeconomically deprived areas of the municipality of Guarulhos [27]. These *UBSs* are primary care clinics that belong to the Brazilian Unified Health System (SUS) and provide primary health care to families registered with FHTs [28]. *Guarulhos* is located in the metropolitan region of *São Paulo* city, has a population of 1.3 million, of which 13.8% are at least 60 years old [29]. Depressive symptomatology has been identified in 30% of the older people of *Guarulhos* [30].

The same inclusion and exclusion criteria of the screening phase of the PROACTIVE trial were used in the present study. Eligible participants for screening were individuals 60 years or older registered with one of the selected FHTs. Exclusion criteria for participating in the screening phase were individuals whose partner—or another person living in the same household—had already been assessed for screening, individuals identified with acute suicidal risk, individuals who had cognitive, communication, visual, or hearing problems, and individuals with terminal illness. We excluded from the analysis of the present study individuals who did not contribute with information about the outcome (postural imbalance), the exposure (depressive symptomatology), or the potential confounders or moderators (described below).

### 2.2 Data Collection

The Guarulhos Health Secretariat provided a list of all adults aged 60 years or over registered in the 20 *UBSs* that took part in PROACTIVE. The list was sorted in random order before trained research assistants began data collection, which took place between May 2019 and February 2020. The screening assessment was carried out at home in a face‐to‐face interview. During the first month of data collection, screening was also conducted by telephone only in a few cases, but it was discontinued for logistic reasons. Written informed consent was obtained from individuals before the screening interview began. In a few cases that the screening interview was conducted by telephone, verbal consent was audio recorded. Detailed information on participant recruitment has been previously published [27]. The study was approved by the Ethics Committee of the *Universidade de São Paulo* Medical School (CEP FMUSP number 2.836.569) and authorised by the *Guarulhos* Health Secretariat.

### 2.3 Measures

#### 2.3.1 Exposure

Depressive symptomatology was assessed using the 9‐item Patient Health Questionnaire (PHQ‐9) [31], which measures depressive symptoms and has good internal consistency in Brazilian older adults [32]. We dichotomised the PHQ‐9 (< 10 or ≥ 10), as scores of 10 or more indicate depressive symptomatology [33].

#### 2.3.2 Outcome

Postural imbalance was assessed by asking the following question: *Do you have impaired postural balance?* For those who reported having postural imbalance, we also asked about falls: *Have you had any falls in the last 6 months due to impaired postural balance?* As falls were only assessed in participants with postural imbalance, we created a variable with three ordered categories to describe the outcome: *no postural imbalance, postural imbalance without falls and postural imbalance with falls.* Falls are a significant adverse outcome of balance impairment; therefore, individuals with postural imbalance were categorised based on whether they had fallen due to this condition. Studies have shown that the subjective assessment of postural imbalance is associated with objective measures of postural balance dysfunction [34], and better at predicting falls among older individuals [35].

#### 2.3.3 Potential Confounders or Moderators

These variables were selected from the PROACTIVE screening database before starting the analyses. The choice of variables was based on known risk factors for falls in older adults [18, 19]. We also selected variables indicating the presence of common health problems in this population. The socioeconomic and demographic data collected were gender, age, education (none, 1–4, 5–8 or > 8 years of formal education) and personal income (≤ 1 Brazilain minimum wage, > 1–2 minimum wages, > 2–3 minimum wages, or > 3 minimum wages per month). We also looked at whether participants were employed (part‐time or full‐time) or doing something for which they received an income. To obtain a measure of subjective age (SA), we asked participants: *In general, or most of the time, how old do you feel you are?* Responses were compared to their actual age in a SA discrepancy score, calculated using the expression [(SA − real age)/real age] × 100 [36]. Scores below −75.00 and above +75.00 were recoded as −75.00 and +75.00, respectively [37]. Thus, scores ranged from −75.00 to +75.00, with negative scores indicating feeling younger than one's chronological age and positive scores indicating feeling older. For example, a SA discrepancy score of −15.00 indicates that a participant feels 15% younger than he or she is.

Participants were asked if they had hypertension or diabetes. In the case of a positive answer, it was investigated for how long they had been living with the disease. They were also asked if they experienced shortness of breath when walking, climbing stairs or when the temperature changed. Irrespective of whether people had depressive symptomatology (PHQ‐9 ≥ 10 or < 10), they were asked whether they were currently taking medication for the treatment of depression.

### 2.4 Statistical Analyses

We estimated the overall prevalence of individuals who reported postural imbalance, with its 95% confidence interval (CI), followed by the prevalences of participants who had postural imbalance *with* or *without* falls in the previous 6 months. Mixed effects ordinal logistic regression models were used to quantify the association between the exposure (PHQ‐9 ≥ 10) and confounders or moderators, and the outcome (postural imbalance) using unadjusted and adjusted odds ratios (ORs) with 95% CIs. Variables significantly associated with the outcome in unadjusted models were entered into a final adjusted model in a single step. The categories of the ordered outcome variable were arranged in the following order: no postural imbalance, postural imbalance *without* falls and postural imbalance *with* falls (highest). The major assumption underlying ordered logistic regression is that the effect of the independent variables remains constant for each increase in the level of the response. This assumption was tested using the *Brant* test in Stata.

Potential confounders or moderators significantly associated with the outcome in unadjusted models were checked for pairwise interactions, either among themselves or between them and the exposure. Only significant interactions that could be justified on a theoretical basis were included in the final adjusted model. Significance of the interaction term was evaluated by command *testparm* in Stata. All regression analyses used random‐effects models with an independent structure of the covariance matrix of the random effects to account for clustering. The primary care clinics participating in the PROACTIVE were the only random effect (clusters). Exposure, outcome and confounders or moderators entered the final model as fixed effects. All analyses were carried out in Stata/IC 15.0 for Windows.

## 3 Results

Of the 3356 older individuals screened for the PROACTIVE randomised controlled trial, 2999 had complete data for the exposure (depressive symptomatology), the outcome (postural imbalance), and the potential confounders or moderators, and therefore were included in the analyses. Among the 2999 participants, 1908 (63.6%) were women, 1895 (63.2%) had up to 4 years of formal education, 1999 (66.7%) earned up to one minimum wage per month, and only 505 (16.8%) had a paid job (see Table 1). Mean age of those participants was 68.3 years. On average, they had a perception of being around 13 years younger than their chronological age. The prevalence of participants with depressive symptomatology, hypertension, diabetes, and shortness of breath was 915 (30.5%), 1955 (65.2%), 991 (33.0%) and 1247 (41.6%) respectively. The current use of medication to treat depression was reported by 238 (7.9%) participants (see Table 1).

**TABLE 1:** Socioeconomic, demographic and health‐related variables of included participants overall and according to the categories of postural imbalance (*N* = 2999).

Variable	Overall (*N* = 2999)	Outcome category		
		No postural imbalance (*N* = 1816)	Postural imbalance without falls (*N* = 792)	Postural imbalance with falls (*N* = 391)
Exposure				
Depressive symptomatology, no. (%)				
No (PHQ‐9 < 10)	2084 (69.5)	1478 (81.4)	441 (55.7)	165 (42.2)
Yes (PHQ‐9 ≥ 10)	915 (30.5)	338 (18.6)	351 (44.3)	226 (57.8)
Potential confounders or moderators				
Gender, no. (%)				
Male	1091 (36.4)	739 (40.7)	253 (31.9)	99 (25.3)
Female	1908 (63.6)	1077 (59.3)	539 (68.1)	292 (74.7)
Education, no. (%)				
None	479 (16.0)	254 (14.0)	145 (18.3)	80 (20.5)
1–4 years	1416 (47.2)	831 (45.8)	381 (48.1)	204 (52.2)
5–8 years	689 (23.0)	441 (24.3)	179 (22.6)	69 (17.6)
> 8 years	415 (13.8)	290 (16.0)	87 (11.0)	38 (9.7)
Personal income in MW, no. (%)				
≤ 1 per month	1999 (66.7)	1136 (62.6)	576 (72.7)	287 (73.4)
> 1–2 per month	626 (20.9)	412 (22.7)	144 (18.2)	70 (17.9)
> 2–3 per month	220 (7.3)	160 (8.8)	44 (5.6)	16 (4.1)
> 3 per month	154 (5.1)	108 (5.9)	28 (3.5)	18 (4.6)
Paid job, no. (%)				
No	2494 (83.2)	1458 (80.3)	692 (87.4)	344 (88.0)
Yes	505 (16.8)	358 (19.7)	100 (12.6)	47 (12.0)
Current use of medication for depression, no. (%)				
No	2761 (92.1)	1720 (94.7)	709 (89.5)	332 (84.9)
Yes	238 (7.9)	96 (5.3)	83 (10.5)	59 (15.1)
Hypertension duration, no. (%)				
Without hypertension	1044 (34.8)	729 (40.1)	208 (26.3)	107 (27.4)
≤ 1 year	135 (4.5)	86 (4.7)	32 (4.0)	17 (4.3)
> 1–5 years	338 (11.3)	209 (11.5)	83 (10.5)	46 (11.8)
> 5–10 years	336 (11.2)	197 (10.8)	93 (11.7)	46 (11.8)
> 10 years	1146 (38.2)	595 (32.8)	376 (47.5)	175 (44.8)
Diabetes duration, no. (%)				
Without diabetes	2008 (67.0)	1297 (71.4)	480 (60.6)	231 (59.1)
≤ 1 year	127 (4.2)	71 (3.9)	39 (4.9)	17 (4.3)
> 1–5 years	272 (9.1)	158 (8.7)	76 (9.6)	38 (9.7)
> 5–10 years	179 (6.0)	105 (5.8)	46 (5.8)	28 (7.2)
> 10 years	413 (13.8)	185 (10.2)	151 (19.1)	77 (19.7)
Shortness of breath, no. (%)				
No	1752 (58.4)	1231 (67.8)	383 (48.4)	138 (35.3)
Yes	1247 (41.6)	585 (32.2)	409 (51.6)	253 (64.7)
SA discrepancy score, mean (SD)	−13.2 (21.2)	−15.5 (20.6)	−10.1 (21.1)	−8.4 (22.9)

*Note:* In the outcome category, falls refer to those due to impaired postural balance. In 2019, the minimum wage in Brazil was 998 Brazilian real (approximately US$253 dollars).

Abbreviations: MW, minimum wage; PHQ‐9, 9‐item Patient Health Questionnaire; SA, subjective age; SD, standard deviation.

Postural imbalance was reported by 1183 participants (39.4%; 95% CI: 37.7%, 41.2%). Of these, 792 (67.0%) reported no falls (non‐fallers), while 391 (33.1%) reported they had fallen in the previous 6 months (fallers). Therefore, of the total number of participants (*N* = 2999), the prevalence of participants who had postural imbalance *without* falls was 26.4% (95% CI: 24.9%, 28.0%) and *with* falls it was 13.0% (95% CI: 11.9%, 14.3%).

Table 2 shows unadjusted and adjusted odds ratios quantifying the association between socioeconomic, demographic and health‐related variables and postural imbalance. Since all variables were significantly associated with postural imbalance in unadjusted models, they were included in the final adjusted model. The association between depressive symptomatology (PHQ‐9 ≥ 10) and the outcome (postural imbalance) in the final model was adjusted for gender, education, personal income, having a paid job, current use of medication for depression, hypertension duration, diabetes duration, shortness of breath and subjective age. We did not find any significant and theoretically justifiable pairwise interaction of variables described as potential confounders or moderators, either among themselves or between them and the exposure.

**TABLE 2:** Unadjusted and adjusted mixed effects ordinal logistic regression analysis for the association of socioeconomic, demographic and health‐related variables and postural imbalance (*N* = 2999).

Variable	Unadjusted OR (95% CI)	*p*	Adjusted OR (95% CI)	*p*
Exposure				
Depressive symptomatology (ref. no [PHQ < 10])				
Yes (PHQ‐9 ≥ 10)	4.07 (3.48; 4.75)	0.000	2.88 (2.44; 3.40)	0.000
Potential confounders or moderators				
Gender (ref. male)				
Female	1.65 (1.41; 1.92)	0.000	1.16 (0.98; 1.39)	0.088
Education (ref. none)				
1–4 years	0.80 (0.66; 0.98)	0.033	0.82 (0.67; 1.02)	0.073
5–8 years	0.62 (0.49; 0.78)	0.000	0.71 (0.55; 0.90)	0.005
> 8 years	0.49 (0.37; 0.64)	0.000	0.63 (0.47; 0.84)	0.002
Personal income in MW (ref. ≤ 1 per month)				
> 1–2 per month	0.70 (0.58; 0.84)	0.000	0.95 (0.78; 1.16)	0.618
> 2–3 per month	0.50 (0.36; 0.67)	0.000	0.81 (0.58; 1.13)	0.217
> 3 per month	0.58 (0.41; 0.83)	0.003	0.96 (0.65; 1.41)	0.842
Paid job (ref. no)				
Yes	0.59 (0.48; 0.73)	0.000	0.84 (0.67; 1.05)	0.121
Current use of medication for depression (ref. no)				
Yes	2.44 (1.90; 3.13)	0.000	1.44 (1.10; 1.87)	0.007
Hypertension duration (ref. without hypertension)				
≤ 1 year	1.31 (0.90; 1.89)	0.154	0.96 (0.65; 1.42)	0.837
> 1–5 years	1.42 (1.10; 1.82)	0.007	1.24 (0.95; 1.62)	0.117
> 5–10 years	1.59 (1.24; 2.04)	0.000	1.15 (0.88; 1.51)	0.293
> 10 years	2.03 (1.71; 2.41)	0.000	1.36 (1.13; 1.64)	0.001
Diabetes duration (ref. without diabetes)				
≤ 1 year	1.41 (0.99; 1.99)	0.055	1.21 (0.83; 1.76)	0.316
> 1–5 years	1.32 (1.03; 1.70)	0.030	1.16 (0.88; 1.51)	0.288
> 5–10 years	1.33 (0.98; 1.81)	0.063	1.23 (0.89; 1.70)	0.212
> 10 years	2.10 (1.72; 2.57)	0.000	1.63 (1.31; 2.02)	0.000
Shortness of breath (ref. no)				
Yes	2.75 (2.37; 3.18)	0.000	1.94 (1.66; 2.28)	0.000
SA discrepancy score (continuous)	1.01 (1.01; 1.02)	0.000	1.01 (1.00; 1.01)	0.005

*Note:* Categories of postural imbalance were arranged in the following order: no postural imbalance (lowest), postural imbalance without falls, and postural imbalance with falls (highest). Adjusted model included all variables. All models used random‐effects with an independent structure of the covariance matrix to account for clusters (primary care clinics). In 2019, the minimum wage in Brazil was 998 Brazilian real (approximately US$253 dollars). Missing values: the following variables had missing values (number and percentage out of *N* = 3356 are displayed): postural imbalance (*n* = 61 [1.8%]), education (*n* = 20 [0.6%]), personal income (*n* = 210 [6.3%]), pid job (*n* = 19 [0.6%]), current use of medication for depression (*n* = 23 [0.7%]), hypertension duration (*n* = 22 [0.7%]), diabetes duration (*n* = 27 [0.8%]), shortness of breath (*n* = 25 [0.7%]), and subjective age (*n* = 81 [2.4%]).

Abbreviations: CI, confidence interval; MW, minimum wage; OR, odds ratio; PHQ‐9, 9‐item Patient Health Questionnaire; SA, subjective age.

We reported the proportional odds ratio comparing participants with depressive symptomatology to those without it. In the adjusted final model, for older adults with depressive symptomatology, the odds of having postural imbalance *with* falls combined to postural imbalance *without* falls versus not having postural imbalance is 2.88 (95% CI: 2.44, 3.40) times that of older adults without depressive symptomatology, holding constant all other variables. Similarly, for older adults with depressive symptomatology, the odds of having postural imbalance *with* falls versus having postural imbalance *without* falls combined to not having postural imbalance is 2.88 (95% CI: 2.44, 3.40) times that of older adults without depressive symptomatology, holding constant all other variables. The parallel odds assumption held (*Brant* test *p* = 0.228). These results are in line with our hypothesis that postural imbalance is more frequent in people with depressive symptomatology than those without it and, among those with postural imbalance, those with depressive symptomatology present more falls than those without depressive symptomatology.

## 4 Discussion

Postural imbalance was reported by 39.4% of participants, and of those reporting postural imbalance, one‐third reported falls associated with imbalance. We found a strong, positive association between depressive symptomatology and postural imbalance: older adults identified with depressive symptomatology (PHQ‐9 ≥ 10) had increased odds of reporting postural imbalance and postural imbalance with falls. This association was independent of gender, education, personal income, having a paid job, current use of medication for depression, hypertension duration, diabetes duration, shortness of breath and subjective age.

Nationwide studies that assessed self‐reported balance problems found lower rates than in our population. Although these studies investigated balance problems in the previous 12 months (rather than six), the definition of balance problems was broader than in our study, as they did not differentiate between impaired balance or dizziness or walking problems [1, 38]. A Swedish twin registry of adults aged 55 years or over found a 12% prevalence of self‐reported impaired balance at the interview [39]. It is worth noting that the sample had a high fitness level (72% reported exercising moderately to intensely), which may have contributed to the relatively low rate. Two large epidemiological studies that identified balance disorders, one conducted in England and the other in the Municipality of Sao Paulo, Brazil (mean age: 69.6 years), also found lower prevalences of balance impairment than in our study, respectively 21.5% [23] and 16.3% [2]. In these studies, individuals from lower socioeconomic backgrounds, who presents a higher frequency of multimorbidity [40], were underrepresented, which is not the case in our sample where 66.7% of participants lived on a monthly income of one minimum wage (approximately US$253) or less. Also, those studies used a battery of balance tests, while we used a self‐reported measure to identify balance impairment. These considerations may explain the relatively high rate of postural imbalance we found.

The most common causes of falls in older people are environmental, that is accidents. Although balance disorders are an important cause of falls, on average only 17% of falls in older individuals are due to gait or postural balance problems [41], an estimate closer to our findings. Pooled data from three large studies showed a 6‐month prevalence of self‐reported falls of 34.3% in community‐dwelling older Brazilians [42], which is higher than in our study. The discrepancy in the results may possibly be because we only looked at falls due to impaired postural balance while those studies were interested in all types of falls, regardless of their cause.

Our findings support a positive association between depressive symptomatology and postural imbalance. Postural balance problems can be manifestations of several latent diseases [10, 11], yet they are often neglected and under‐assessed in clinical practice, especially in primary care [43]. Early identification and treatment of balance problems can help prevent falls and also improve functional ability, independence and quality of life in older age [10, 25], which are well known risk factors for depression. In the same way as fall prevention initiatives and interventions for depressive symptoms [25, 26], detection of postural imbalance should be adapted to the level of resources available in primary care to help save resources, especially in LMICs.There are some limitations of this work. First, our study did not include individuals of higher socioeconomic status. These people commonly use private health care and live in areas of *Guarulhos* not covered by FHTs. The FHTs reach remote and socioeconomically disadvantaged areas of Brazil, which tend to be the least supported by other health services. However, our sample has characteristics similar to most Brazilian older adults, who are poor and covered by FHTs [44]. Second, the analysis included only characteristics of participants assessed at the screening phase of the PROACTIVE trial. As a result, conditions that affect balance functioning, such as Parkinson's disease, were not added to the regression models. Nonetheless, these medical conditions are infrequent in the older Brazilian population [45]. Third, data on postural balance impairment was self‐reported rather than objectively measured using a validated balance test. Despite the lack of data regarding the positive and negative predictive value of balance complaints in identifying objective balance impairment among older adults, self‐reported measures of imbalance have been demonstrated to be strongly associated with performance in balance tests [34]. Indeed, recent evidence suggests that self‐perceived balance impairment is a better predictor of falls than objective measures of imbalance [35]. The advantage of self‐reported measures is that they are simple and easy to obtain, especially in health services with limited resources, and may encompass a history of balance symptoms, occurred long before the data collection, which may not be captured in a single balance test [46]. Falls investigated in this study were due to impaired postural balance, which made it difficult to compare with previous studies that evaluated falls resulting from a variety of causes. Finally, this study does not allow the identification of causality of associations because all the data were collected simultaneously. Thus, longitudinal studies are necessary to explain better the relationship between depression and postural imbalance in community‐dwelling older people. To our knowledge, no such research has been conducted in LMICs. In addition, and bearing in mind the exploratory nature of our analyses, our findings need to be replicated in other samples.

## 5 Conclusion

We analysed a large sample of older adults living in a socioeconomically deprived area in Brazil, registered in primary care clinics. This study contributes to the understanding of the relationship between depressive symptomatology and postural imbalance, which has been inconclusive and understudied to date.

## Ethics Statement

Written informed consent was obtained from all participants before starting the study. If the screening assessment was made by telephone, verbal consent was audio recorded. The study was approved by the Ethics Committee of the *Universidade de São Paulo* Medical School (CEP FMUSP number 2.836.569) and authorised by the *Guarulhos* Health Secretary.

## Conflicts of Interest

The authors declare no conflicts of interest.

## Data Availability

The data that support the findings of this study are available from the corresponding author upon reasonable request.
